# c-di-GMP-mediated pause behavior enables *Pseudomonas aeruginosa* navigation in porous environments

**DOI:** 10.1128/aem.00597-26

**Published:** 2026-04-27

**Authors:** Zihuan Zhang, Rongjing Zhang, Junhua Yuan

**Affiliations:** 1 Hefei National Research Center for Physical Sciences at the Microscale and Department of Physics, University of Science and Technology of China 12652, Hefei, Anhui, China; Indiana University Bloomington, Bloomington, Indiana, USA

**Keywords:** bacterial motility, porous environments, mechanosensing, c-di-GMP

## Abstract

Bacteria frequently encounter porous environments in nature and clinical settings, yet how single-flagellated pathogens navigate such confined spaces remains unclear. Here, we investigate *Pseudomonas aeruginosa* motility in soft agar using three-dimensional particle tracking. We discover that *P. aeruginos*a exhibits a distinct “pause” motility mode characterized by sustained low-speed motion with continuous reorientation in addition to its characteristic run-and-reverse swimming pattern. During pauses, flagellar filaments actively swing between adjacent pore openings while the cell body remains confined, facilitating reorientation and escape. Remarkably, bacteria pre-incubated in agar continue exhibiting pause behavior after transfer to bulk liquid, indicating that porous environments trigger lasting physiological adaptations beyond physical confinement. We demonstrate that this behavioral plasticity is mediated by elevated intracellular cyclic di-GMP levels and requires the PilZ domain effector FlgZ. These findings reveal how bacteria integrate mechanical environmental cues into regulatory circuits controlling locomotion, providing insights into pathogen behavior in clinically relevant porous environments.

**IMPORTANCE:**

Bacterial pathogens must often navigate complex, confined spaces, such as tissues, mucus, and medical materials. However, how single-flagellated bacteria like *Pseudomonas aeruginosa* adapt their motility strategies to porous environments remains poorly understood. Here, we reveal that *P. aeruginosa* exhibits a distinct “pause” motility mode when encountering pore confinement, characterized by repeated reorientation events that facilitate escape. Remarkably, this behavior persists even after bacteria are returned to liquid, indicating a physiological memory triggered by the environment. We demonstrate that this adaptive response is regulated by the second messenger c-di-GMP and its effector FlgZ. Our findings uncover a direct link between mechanical environmental sensing and the molecular regulation of bacterial motility, providing new insights into how pathogens adapt to and persist in clinically and environmentally relevant porous habitats.

## INTRODUCTION

Bacteria frequently encounter complex porous environments in nature and clinical settings. Understanding bacterial motility through such confined spaces has important implications for human health, environmental microbiology, and biotechnology. The opportunistic pathogen *Pseudomonas aeruginosa* provides a compelling example, as it navigates diverse three-dimensional matrices throughout its lifecycle: from penetrating the protective mucus barriers of cystic fibrosis patients (1, 2) to migrating through soil micropores during environmental persistence (3), infiltrating groundwater systems (4), and contaminating food matrices (5, 6). Understanding how bacteria adapt their locomotion strategies to these confined geometries is therefore important for predicting pathogen spread, designing antimicrobial interventions, and engineering biotechnological applications.

While bacterial motility in bulk liquids has been well characterized, movement through realistic porous environments remains less understood. This is especially true for single-flagellated bacteria like *P. aeruginosa*, which employ propulsion mechanisms distinct from those of the well-studied multi-flagellated model organism *Escherichia coli*. In *E. coli*, the coordination of 4–6 peritrichous flagella enables the canonical run-and-tumble motility pattern, where bundled flagella propel forward motion during runs, while flagellar unbundling triggers random reorientation during tumbles (7–9). Recent studies have shown that porous media alter this behavior: *E. coli* cells become transiently trapped in narrow pores, where flagellar unbundling is suppressed until reorientation enables escape (10).

In contrast, *P. aeruginosa* possesses a single polar flagellum (11) that generates different motility patterns, including run-reverse-pause (12, 13) or run-reverse-wrap (14, 15) behavior in bulk liquid. These architectural and behavioral differences raise questions about how single-flagellated bacteria navigate porous environments and whether they employ adaptive strategies distinct from multi-flagellated species. Notably, *P. aeruginosa* also possesses complex signal transduction networks, including cyclic di-GMP (c-di-GMP) systems that integrate environmental cues to modulate cellular behavior (16). Whether these regulatory networks contribute to motility adaptation in confined spaces remains to be explored.

The best-characterized context for c-di-GMP-mediated behavioral adaptation in *P. aeruginosa* is the initial encounter with a surface, which can trigger the transition from planktonic motility to surface-attached biofilm communities (17). This transition is mediated in part by mechanosensory systems that detect surface contact and transduce these signals into changes in intracellular c-di-GMP levels. The Wsp system, a chemosensory pathway homologous to the Che chemotaxis system, is activated by cell envelope stress associated with surface growth (18). Upon stimulation, the chemoreceptor WspA initiates a phosphorylation cascade that activates the diguanylate cyclase WspR, leading to localized production of the second messenger c-di-GMP (19). In parallel, the Pil-Chp system transduces mechanical signals detected by type IV pili (T4P). The adhesin PilY1, located at the pilus tip, senses mechanical stimuli, such as shear forces, during surface contact (20). Upon T4P engagement with a surface, PilY1 signals through the PilJ–ChpA two-component system to activate the adenylyl cyclase CyaB, producing cAMP, and through the T4P alignment complex PilMNOP to activate the diguanylate cyclase SadC, producing c-di-GMP. Together, these pathways converge on c-di-GMP, a ubiquitous second messenger that regulates diverse phenotypes in *P. aeruginosa*, including motility, biofilm formation, antibiotic resistance, and virulence (21). The downstream effects of c-di-GMP are mediated by binding to a variety of effector proteins, including PilZ domain proteins, catalytically inactive diguanylate cyclases and phosphodiesterases, riboswitches, and transcription factors (22). While these surface-sensing pathways have been extensively studied in the context of two-dimensional surface attachment, whether analogous mechanosensory signaling is activated when cells navigate through three-dimensional porous matrices—where they experience confinement, increased drag, and physical contact with surrounding structures—has not been investigated.

Here, we employ three-dimensional single-cell tracking to investigate how *P. aeruginosa* navigates porous environments, using soft agar as a well-controlled model system with tunable pore size distributions (23, 24). Our results reveal that porous confinement induces a previously unrecognized motility mode characterized by prolonged cellular reorientation, which we term “pause” behavior. Interestingly, this adaptive response persists even after cells are returned to bulk liquid, suggesting an underlying physiological memory mechanism. We demonstrate that this behavioral plasticity is mediated by the c-di-GMP signaling network, specifically through the PilZ domain effector FlgZ, revealing how bacteria integrate mechanical environmental cues into regulatory circuits that control locomotion. These findings provide insights into bacterial adaptation to complex environments and have implications for understanding pathogen behavior in clinically relevant settings.

## RESULTS

### Characterization of pore size distributions in soft agar

Soft agar, a gel composed primarily of crosslinked agarose molecules, forms a porous network upon cooling from a molten state (23). The pore sizes within soft agar follow a normal distribution, with mean pore diameter decreasing as agar concentration increases (24). Its stability and biocompatibility make soft agar an ideal model system for studying bacterial motility in disordered three-dimensional porous environments. We characterized pore size distributions by tracking the Brownian motion of 350-nm beads, where the mean square displacement (MSD) at long lag times provides a measure of pore confinement (25). Briefly, the mean square displacement (MSD) of an individual d=350nm bead in a certain pore reaches a plateau ($MSD_{\infty }$) at long lag times (Fig. S1). The size of this pore is determined by $d+{\left(MSD_{\infty }\right)}^{1/2}$. Pore size distributions were obtained by tracking many beads (Fig. S2). The mean pore diameters were approximately 1.79 ± 0.73 µm for 0.05% agar, 1.56 ± 0.34 µm for 0.1% agar, 1.14 ± 0.69 µm for 0.15% agar, and 0.72 ± 0.38 µm for 0.2% agar, confirming that increasing agar concentration produces progressively smaller pores.

### *P. aeruginosa* exhibits a novel pause motility mode in porous media

**Fig 1:**
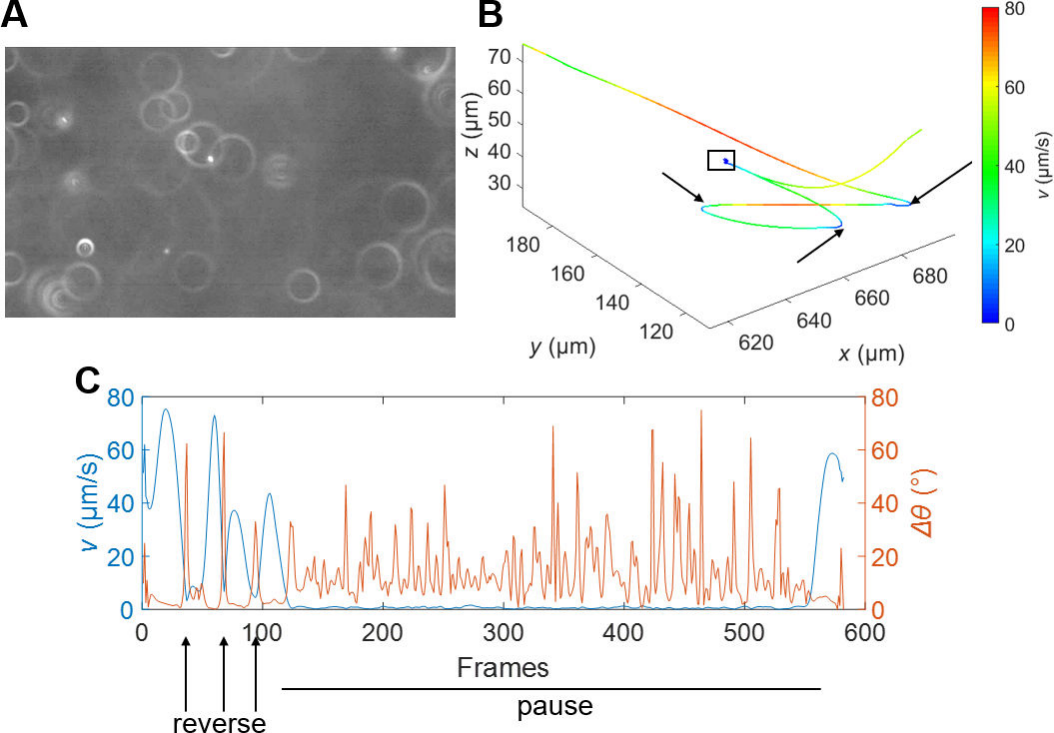
*P. aeruginosa* exhibits three distinct motility modes in soft agar. (**A**) Representative frame from 3D tracking experiments showing *P. aeruginosa* cells as bright rings in dark-field microscopy. (**B**) Typical 3D trajectory of a single bacterium in soft agar. The color bar indicates the instantaneous speed of the bacterium. The trajectory exhibits extended directional swimming (runs), abrupt directional changes (reverses, arrows), and prolonged low-speed reorientation events (pause, boxed region). (**C**) Quantitative analysis of the trajectory in (**B**) showing instantaneous speed and angular change between consecutive frames as functions of time. Arrows indicate reverse events; horizontal line denotes the pause period characterized by sustained low speed and repeated directional changes.

Having established the pore size distributions in soft agar, we next examined how *P. aeruginosa* navigates these confined environments at the single-cell level. We tracked *P. aeruginosa* in three dimensions within LB soft agar using defocused particle tracking based on dark-field microscopy. In this technique, each cell appears as a bright ring, with the ring radius correlated to the defocusing distance from the focal plane. An example image is shown in Fig. 1A. By calibrating the relationship between the ring radius *r* and the defocusing distance *z*, we determined the *x*, *y,* and *z* coordinates of individual cells over time from the center and radius of the bright rings, enabling reconstruction of complete three-dimensional trajectories. Trajectories longer than 3 s were selected for further analysis to ensure sufficient data points for reliable classification of motility modes.

Analysis of individual trajectories revealed that *P. aeruginosa* exhibits three distinct motility modes in soft agar. In addition to the expected run and reverse swimming behaviors observed in bulk liquid, cells displayed a third mode characterized by entrapment within pore structures. Figure 1B shows a representative trajectory where a cell executed three run-reverse cycles (arrows) before becoming trapped (boxed region) for approximately 30 s, after which it escaped and resumed running.

To quantitatively distinguish these motility modes, we analyzed instantaneous swimming speed (*v*) and the angular change in velocity between consecutive frames (∆θ) as functions of time. During runs, cells maintain relatively high speeds with minimal directional changes. Reverse events are characterized by low speeds and a single large directional change. In contrast, the newly identified mode—which we term “pause”—involves sustained low speeds accompanied by multiple directional changes as cells undergo repeated reorientation attempts.

We developed a classification algorithm based on these kinematic signatures. From individual trajectories, we identified speed minima and calculated the local speed decrease Δ*v* relative to flanking speed maxima. A candidate event was identified when Δ*v* exceeded both 1.5 times the local minimum speed and a fixed threshold of 9 μm/s. The choice of this speed threshold did not significantly affect the resulting distributions of run lengths and pause durations (Fig. S5). Event duration was determined by the interval over which speed remained within 40% of Δ*v* above the local minimum. Candiate events were then classified as pauses if multiple peaks in the angular change Δ*θ* exceeded the expected contribution from rotational diffusion ($2\sqrt{4D_{rot}\Delta t}$, where $D_{rot}=0.1rad^{2}/s$ and Δ*t* = 1/15 s) during the event; otherwise, they were classified as reversals. This distinction captures the key behavioral difference: reversal events feature a single large directional change, whereas pauses involve multiple consecutive reorientation attempts. Full mathematical details are provided in Materials and Methods. Notably, the run-reverse-pause swimming pattern was also observed in agarose gel (Fig. S3), confirming that this behavior is not specific to agar.

**Fig 2:**
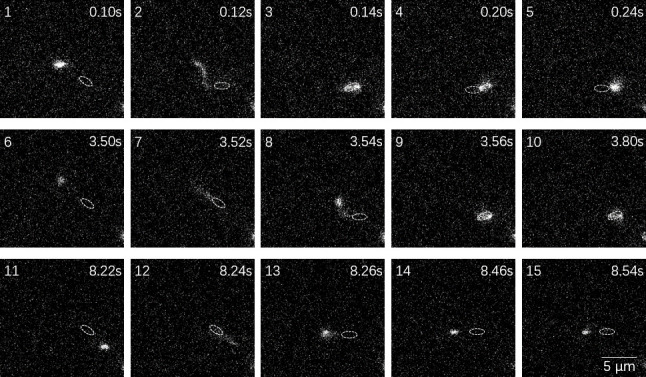
Flagellar filament dynamics during pause behavior in porous media. Time-lapse sequence showing a fluorescently labeled flagellar filament exploring pore space while the cell body remains confined. The flagellar filament exhibits two distinct behaviors: rapid movement between pores (frames 1–3, 6–9, and 11–13) and temporary entrapment within narrow spaces (frames 3–5, 9–10, and 13–15). White dashed ellipses indicate cell body position. Time stamps are shown in the top right corner of each frame. The image sequence was taken from Movie S1. Scale bar, 5 μm.

To understand flagellar behavior during pause events, we fluorescently labeled flagellar filaments in the *fliC^T394C^* strain using a thiol-reactive dye. This strain exhibits motility comparable to wild-type cells in liquid medium (Fig. S4). Direct visualization revealed that during pause mode, the flagellar filament operates independently of the confined cell body (Fig. 2; Movie S1). While the cell body remains nearly stationary, undergoing repeated reorientations consistent with physical confinement by the surrounding gel network, the flagellar filament extends beyond the immediate vicinity of the cell body, exhibiting rapid oscillations or dramatic swinging motions as it probes the adjacent pore spaces.

The occurrence of these specific motility modes is directly linked to the physical parameters of the local environment. While runs and reverses are intrinsic behaviors that persist in unconfined liquid, the pause mode is triggered specifically when a bacterium encounters a pore narrower than its effective escape path, physically arresting forward progress. Consequently, as the mean pore diameter decreases with higher agar concentrations (from 1.79 ± 0.73 µm in 0.05% agar to 0.72 ± 0.38 µm in 0.2% agar), cells encounter these physical bottlenecks more frequently, forcing a transition from running to pausing.

### Physical confinement alters motility statistics and promotes pause-mediated reorientation

**Fig 3:**
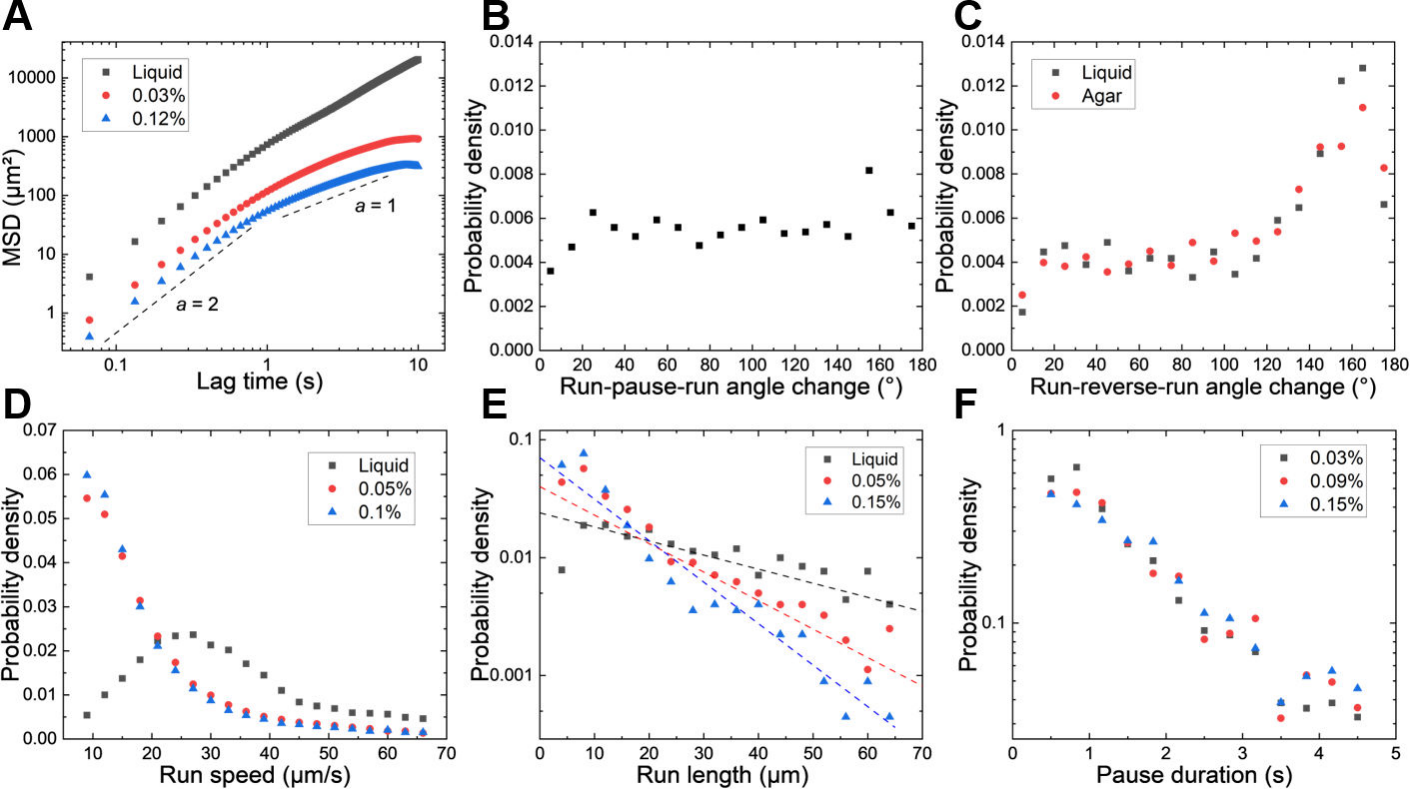
Motility and pause behavior statistics are modulated by agar concentration. (**A**) MSD as a function of lag time for *P. aeruginosa* in bulk liquid and soft agar of varying concentrations. Black dashed lines indicate ballistic (*t*^2^) and normal diffusive (*t*) scaling as visual guides. (**B**) Probability density function of directional changes in run-pause-run transitions (in 0.1% agar). (**C**) Probability density function of directional changes in run-reverse-run transitions in soft agar and bulk liquid. Black and red symbols denote data for liquid and 0.1% agar, respectively. (**D–F**) Distributions of swimming speeds (**D**), run lengths (**E**), and pause durations (**F**) for *P. aeruginosa* in porous media. Legends indicate agar concentrations. Dashed lines in (**E**) represent exponential fits to the corresponding data.

We characterized the diffusive properties of cells through mean square displacement (MSD) analysis. The relationship between MSD and lag time *t* follows the form $MSD=Dt^{a}$, where *D* is the generalized diffusion coefficient, and *α* is the anomalous exponent characterizing the diffusion. As shown in Fig. 3A, at short times, MSD scales as $t^{2}$, indicating ballistic motion. Beyond a critical lag time, the exponent *a* decreases below 1, indicating subdiffusive behavior. This subdiffusion resembles Knudsen diffusion observed in gas molecules when the mean free path becomes comparable to the characteristic pore diameter (26).

To investigate the role of pause events in cellular reorientation, we compared directional changes between adjacent runs in run-reverse-run versus run-pause-run transitions. Directional changes following pause events are broadly distributed across all angles (Fig. 3B), whereas direction changes following reverse events are narrowly concentrated between 140° and 180° (Fig. 3C). These observations suggest that directional changes in run-pause-run transitions are influenced by the physical structure of the gel, where free paths are randomly oriented in space. In contrast, reversal is an intrinsic swimming mode, and the directional changes in run-reverse-run transitions for *P. aeruginosa* in soft agar remain consistent with those observed in liquid environments (14). Liquid data are absent from Fig. 3B and F because pauses account for less than 1% of motility events in bulk liquid, yielding insufficient statistics for meaningful distributions of either run-pause-run angle change or pause duration.

We next analyzed the distributions of run speeds (Fig. 3D), run lengths (Fig. 3E), and pause durations (Fig. 3F). In bulk liquid, *P. aeruginosa* swimming speeds peak at approximately 30 μm/s. However, in soft agar, the probability density decreases monotonically with increasing speed. Run length distributions show faster decay in denser agar, indicating more frequent interruptions. Pause durations follow exponential distributions that remain largely independent of agar concentration. These results indicate that agar constrains bacterial motility primarily by reducing swimming speeds and shortening run lengths, while pause duration appears to be governed by intrinsic cellular mechanisms rather than pore geometry.

### One-hour exposure to soft agar downregulates motility of *P. aeruginosa*

Having observed that *P. aeruginosa* cells exhibit pause behavior in agar gel, we investigated whether this mode results solely from physical pore confinement or if it also involves physiological effects. To distinguish between these mechanisms, we designed an experiment to minimize physical gel constraints while preserving potential physiological responses. We uniformly embedded log-phase *P. aeruginosa* cells in bulk soft agar of varying concentration (by mixing cells with molten agar at 37°C and allowing the mixture to gel at room temperature) for 1 h. We then mechanically disrupted the soft gel by gentle pipetting, washed the cells via centrifugation, and resuspended them in fresh liquid LB broth. This treatment removed physical agar confinement while potentially preserving any cellular responses induced by agar exposure.

**Fig 4:**
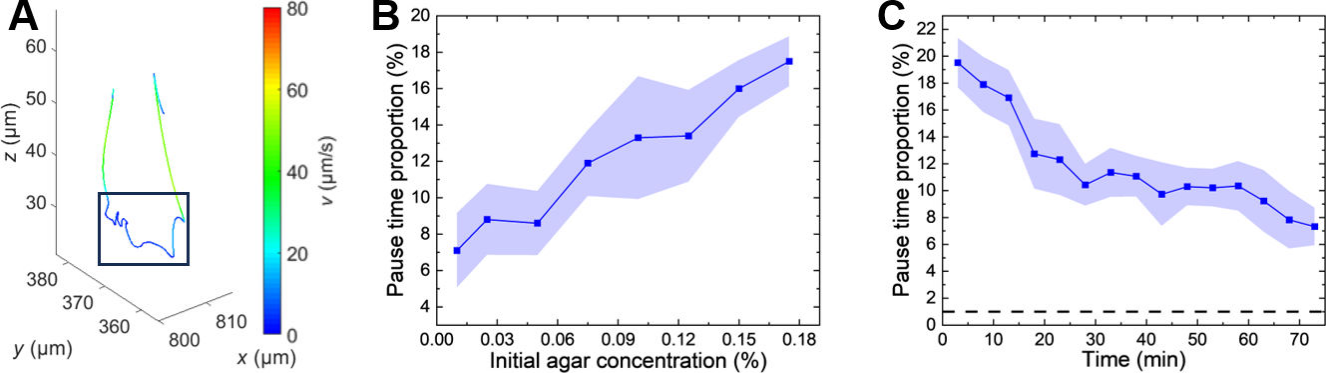
Pause behavior persists in *P. aeruginosa* following agar exposure and transfer to liquid medium. (**A**) Representative trajectory of a bacterium after transfer from 0.2% agar to liquid LB, showing characteristic pause behavior (boxed region) despite the absence of physical confinement. (**B**) Average pause time proportion during the first 10 min in liquid LB as a function of initial agar concentration used for pre-incubation. (**C**) Time course of pause time proportion following transfer to liquid LB for bacteria pre-incubated in 0.2% agar. Error bars represent standard error of the mean (*n* = 5 biological replicates). Dashed line indicates the pause time proportion of 1%.

Three-dimensional tracking of these agar-exposed cells in liquid medium revealed a behavior remarkably similar to the pause mode observed in gel. As shown in Fig. 4A, bacteria exhibited the characteristic sequence of running, reversing, then undergoing multiple low-speed reorientation events before resuming directed swimming. This pause-like behavior occurred despite the absence of physical pore constraints, suggesting that agar exposure triggers physiological changes in motility regulation.

To quantify this effect, we analyzed the motility of cells pre-incubated in soft agar of varying concentrations and calculated the average proportion of time spent in pause mode within 10 minutes of transfer to liquid LB. The pause time proportion increased with initial agar concentration (Fig. 4B), indicating a dose-dependent response to agar exposure. Moreover, this effect was transient, with pause time proportion decreasing over time and reaching ~7% after approximately 70 min (Fig. 4C, 0.2% agar). The pause time proportion without prior incubation in soft agar is below 1%.

These results demonstrate that soft agar exerts both physical and physiological effects on *P. aeruginosa* motility. The physiological response is concentration-dependent (and thus pore size-dependent), persists for over an hour after agar removal, and suggests that cells can sense and respond to porous environments through mechanisms beyond simple physical obstruction.

### Agar-mediated motility downregulation involves the c-di-GMP effector FlgZ

**Fig 5:**
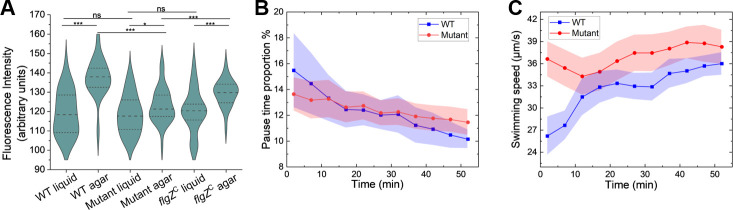
The c-di-GMP effector FlgZ regulates bacterial motility in agar gel. (**A**) Violin plots showing c-di-GMP reporter fluorescence levels for three strains in agar gel and bulk liquid: wild-type, *flgZ*^R126A R130A^ mutant, and *flgZ*^R126A R130A^ mutant complemented with wild-type FlgZ (denoted as *flgZ*^C^). (**B and C**) Time course of pause time proportion (**B**) and average swimming speed (**C**) for wild-type and *flgZ*^R126A R130A^ mutant bacteria following transfer from 0.1% agar to liquid medium. Error bands represent standard error of the mean (*n* = 10 biological replicates). Statistical significance: ***P < 0.001, **P < 0.01, *P < 0.05, ns: *P* > 0.05.

Our observations above suggest that agar exposure induces physiological changes in *P. aeruginosa* motility. Since cyclic-di-GMP (c-di-GMP) is a multifunctional second messenger that links various environmental cues to cellular responses (16), we investigated whether c-di-GMP plays a role in regulating *P. aeruginosa* motility in porous media. Using the pCdrA::*gfp* fluorescent reporter plasmid (27) as an indicator of cellular c-di-GMP levels, we compared fluorescence between cells cultured in LB broth with and without agar. As shown in Fig. 5A, after 1 h of incubation in soft agar, the level of fluorescence (and thus the level of cellular c-di-GMP) increased significantly. These results suggest that agar-induced elevation of c-di-GMP contributes to the pause behavior observed in porous media, consistent with the well-established role of c-di-GMP as an important regulator of *P. aeruginosa* motility.

Elevated c-di-GMP levels can affect bacterial behavior by binding to various effector proteins. Among these effectors, the PilZ domain protein FlgZ has been reported to impede bacterial motility by interacting with the MotCD stator complex (28). To test FlgZ involvement, we mutated its c-di-GMP binding motif (RNAYR to ANAYA) (29). This mutation did not affect bacterial motility in bulk liquid (Fig. S4), and unlike other previously reported non-binding FlgZ variants (30), this specific mutant has been shown to be stably expressed (29).

We then compared motility recovery kinetics between wild-type and FlgZ mutant cells following transfer from agar to liquid LB. Over 50 min post-transfer, wild-type cells showed substantial recovery with pause time proportion decreasing by 35% (from 15.5% to 10.1%) and swimming speeds increasing over time (Fig. 5B and C). In contrast, the FlgZ mutant showed more limited recovery, with pause time proportion decreasing by only 16% (from 13.6% to 11.4%) and swimming speeds remaining relatively constant. Notably, immediately post-transfer, the FlgZ mutant exhibited lower pause time proportion and higher run speeds compared to wild-type cells. These results suggest that FlgZ actively mediates agar-induced motility changes. The effect of *flgZ* mutation on swimming speed (Fig. 5C) is more pronounced than on pause time proportion (Fig. 5B), suggesting that c-di-GMP acts through FlgZ to broadly modulate flagellar motion, with its strongest effect on swimming speed and a more modest contribution to pause behavior. This is consistent with the possibility that additional c-di-GMP effectors or c-di-GMP-independent pathways also contribute to the regulation of pause behavior.

To further investigate this mechanism, we examined c-di-GMP levels in the FlgZ mutant following agar incubation. Previous studies indicate that the FlgZ/c-di-GMP complex can bind the stator protein MotC and activate the diguanylate cyclase SadC, amplifying c-di-GMP production (28). While we observed no significant difference in c-di-GMP levels between wild-type and mutant cells in liquid LB, the mutant showed significantly lower c-di-GMP levels than wild-type in agar medium (Fig. 5A). This reduction is consistent with the lower initial pause time proportion and higher run speeds observed immediately post-transfer in the mutant. These findings suggest that agar-induced elevation of c-di-GMP regulates bacterial motility through FlgZ-mediated pathways. This conclusion was further confirmed by complementing the *flgZ* mutant with a plasmid expressing wild-type FlgZ, which successfully restored the wild-type phenotype (Fig. 5A).

## SUMMARY AND DISCUSSION

In this study, we investigated the motility behavior of *P. aeruginosa* in agar gel using three-dimensional tracking microscopy. Through systematic analysis of bacterial trajectories, we discovered that *P. aeruginosa* maintains its intrinsic run-and-reverse swimming pattern but frequently exhibits a distinct pause behavior when confined within gel pores. During these pauses, flagellar filaments actively swing between adjacent pore openings, likely facilitating cell escape. Run lengths decrease and pause frequency increases with agar concentration, indicating a direct relationship between pore geometry and motility patterns.

To distinguish between physical confinement and physiological adaptation, we incubated bacteria in agar for 1 h before transferring them to liquid medium. Remarkably, these agar-exposed cells continued to exhibit pause behavior in liquid, demonstrating that agar exposure triggers lasting physiological changes beyond simple physical confinement. We further identified elevated intracellular c-di-GMP levels following agar exposure and showed that the PilZ domain effector FlgZ mediates these motility changes, establishing a molecular mechanism linking environmental sensing to behavioral adaptation.

Leveraging dark-field microscopy combined with defocusing particle tracking, we achieved 3D visualization of bacterial motion within porous environments, enabling precise tracking of bacterial positions while preserving conditions close to natural settings. Across all agar concentrations tested, *P. aeruginosa* motility was not solely dictated by gel structure; cells persist in run-reverse swimming unless encountering narrow pores, where they undergo continuous reorientation to navigate escape routes. These experimental observations align with prior theoretical predictions of run-reverse microswimmer diffusivity in porous media (6, 31), confirming ballistic motility at short timescales and subdiffusive behavior over longer durations.

The pause behavior we describe differs fundamentally from previously reported pauses in liquid medium (13). The pauses characterized by Cai et al. involve a sudden near-zero speed followed by continuation in the same swimming direction. In contrast, our observed pauses consist of multiple consecutive directional changes with randomized swimming directions upon resumption, reflecting the random spatial orientation of pore entrances and exits in agar. Upon transfer from soft agar to liquid medium, the elevated cellular c-di-GMP levels sustain these pauses until excess c-di-GMP is degraded.

Notably, the pause time proportion in 0.2% agar was 34.3%, while that immediately after transfer to liquid LB was approximately 20%. Pauses in liquid medium following transfer from soft agar likely reflect motility suppression mediated by c-di-GMP. This suggests that pauses in soft agar consist of two components: one resulting from c-di-GMP action and another from physical entrapment within gel pores. Thus, gel pores physically trap bacteria, inducing pause behavior and triggering an increase in intracellular c-di-GMP levels through potential bacterial mechanosensing mechanisms (32–37). The elevated c-di-GMP further suppresses motility, increasing the likelihood of pauses.

Indeed, the behavior we observe in porous media may share mechanistic parallels with bacterial surface sensing. Upon initial contact with a surface, *P. aeruginosa* undergoes a transient period of reduced motility accompanied by elevated c-di-GMP levels, which can ultimately promote the transition from planktonic to biofilm lifestyles (34–36). Surface sensing in *P. aeruginosa* involves multiple mechanosensory inputs, including increased flagellar motor load (32), obstruction of pilus retraction (33), and activation of the Wsp chemosensory system (34, 35). In porous media, cells confined within narrow pores likely experience analogous mechanical stimuli—increased drag on the flagellar motor and physical contact between the cell body and pore walls—that could activate overlapping signaling pathways. The c-di-GMP elevation and FlgZ-dependent motility suppression we observe are consistent with this interpretation, suggesting that navigation through porous environments may engage the same early surface-sensing circuits that precede biofilm commitment. However, unlike irreversible surface attachment, the pause behavior in porous media is transient and ultimately enables cells to escape confinement, raising the possibility that the intensity or duration of mechanical stimulation determines whether cells resume motility or proceed toward surface adaptation.

c-di-GMP-mediated responses often depend on specific protein-protein interactions. FlgZ, a PilZ domain protein, binds the MotCD stator in a c-di-GMP-dependent manner, regulating swimming (38) and swarming (30) motility. Elevated intracellular c-di-GMP levels also suppress motility in *Escherichia coli* and *Salmonella enterica* (39–44) via YcgR, a FlgZ homolog. Here, we demonstrate that FlgZ actively regulates *P. aeruginosa* motility in soft agar, implicating a signaling pathway whereby porous confinement elevates c-di-GMP, which then binds FlgZ. The FlgZ-c-di-GMP complex performs dual functions: downregulating motility via MotCD stator binding and amplifying c-di-GMP synthesis by interacting with the diguanylate cyclase SadC through MotC.

The *flgZ* mutant bacteria still showed an appreciable c-di-GMP increase in soft agar compared with bulk liquid (Fig. 5A), suggesting that additional pathways contribute to sensing porous environments and initiating c-di-GMP elevation. This initial response is likely amplified through the positive feedback loop involving FlgZ, MotC, and SadC. Further characterization of these upstream signaling mechanisms will be crucial to fully understand how bacteria integrate physical and chemical cues to modulate motility in complex habitats.

## MATERIALS AND METHODS

### Strains and cell culture

Strains used in this study are listed in Table S1. A single colony was isolated and cultured overnight in LB broth (1% tryptone, 0.5% yeast extract, 1% NaCl) on a rotary shaker (200 rpm) at 37°C. Then, 100 µL of overnight culture was diluted into 10 mL fresh LB broth and cultured to mid-log phase (OD_600_ ~ 1). When required, gentamicin was added at 30 µg/mL, and ampicillin was added at 15 µg/mL. To induce protein expression, 0.06% arabinose was added.

### Construction of plasmids and mutants

DNA cloning was performed using PCR according to standard methods. To construct the *flgZ*^R126A R130A^ mutant, approximately 1,000 bp DNA fragments upstream and downstream of the mutation sites were amplified by PCR and assembled with linearized suicide vector pEX18Gm (45) using Gibson assembly (46). The recombinant plasmid was electroporated into *P. aeruginosa* PAO1, and transformants were selected on LB plates containing gentamicin. Mutants were verified by DNA sequencing.

### Preparation of agar gel and cell samples

Agar stock solution (0.5%) was prepared by dissolving agar in LB broth and maintained at 68°C. Before use, the stock solution was diluted with LB broth to the desired concentration and cooled to approximately 37°C. Log-phase bacterial cultures were gently mixed into the agar solution, and samples were cooled to room temperature (23°C) to induce polymerization before analysis.

For experiments requiring the extraction of cells from soft agar, the entire volume of the fragile soft agar matrix was mechanically disrupted by gentle pipetting. The suspension was then centrifuged to pellet the cells, effectively separating them from the disrupted agar network, and the pellet was resuspended in fresh liquid LB medium. This complete mechanical disruption ensures high recovery efficiency of the uniformly embedded population.

### Three-dimensional tracking

All microscopy experiments were carried out at a room temperature of 23°C. We used the defocused particle tracking technique, as described previously (47). To calibrate the relationship between the ring radius *r* and the defocusing distance *z*, 1-μm-diameter polystyrene beads were mounted on a glass slide. The height of the slide was adjusted to bring the beads into focus, then beads were displaced vertically in 1-µm increments while recording images at each position. Images at various *z* positions were processed to determine ring radii *r*, and the *z* versus *r* relationship was determined by linear fitting.

For tracking bacterial swimming in three-dimensional porous media, a sample chamber was constructed using two coverslips (18 × 18 mm and 24 × 60 mm) separated by two strips of 1-mm-thick double-sided tape. Cell suspension was injected into the chamber via capillary action. Dark-field microscopy was performed using a Nikon Eclipse Ti-2E microscope equipped with a Ph4 ring slit and Nikon Plan Fluor 20× 0.6 NA objective lens. Videos were recorded at 15 fps using a Thorlabs USB 3.0 digital camera.

### Flagella fluorescence labeling

Flagellar filaments were fluorescently labeled following the protocol described previously (48). Log-phase culture (1 mL) was centrifuged at 1,000 × *g* for 10 min and washed twice with 1 mL motility buffer (50 mM potassium phosphate, 0.15 M NaCl, 15 µM EDTA, 10 mM lactic acid, pH 7.0). The final pellet was resuspended to 300 µL. Alexa Fluor 568 maleimide (Invitrogen-Molecular Probes) was added to a final concentration of 20 µg/mL, and labeling proceeded for 30 min at room temperature with gentle rotation at 80 rpm. Unbound dye was removed by washing cells three times with motility buffer, and cells were resuspended in motility buffer for analysis.

### Fluorescence imaging

A fluorescence chamber was constructed using an 18 × 18 mm coverslip and a glass slide separated by two strips of thin double-sided tape, resulting in a chamber height of about 0.2 mm. The chamber was filled with cell suspension containing fluorescently labeled strains and sealed with Apiezon vacuum grease. Cells were observed using a Nikon Ti-E inverted microscope equipped with Nikon fluorescence filter cubes of appropriate wavelengths, a Nikon Plan Apo 100× 1.45 NA oil-immersion objective lens, and a scientific CMOS camera (Prime95B; Photometrics). Videos were recorded at 50 fps. In fluorescence imaging, the tip of the *P. aeruginosa* flagellum appeared brightest (Fig. 2), likely because sulfhydryl groups on cysteine residues of flagellin are more exposed at the filament tip than along the rest of the filament. These exposed sites have higher affinity for maleimide-tagged fluorescent dye molecules, resulting in enhanced fluorescence at the flagellar tip.

### Data processing

For 3D tracking, videos of bacteria were processed using custom MATLAB scripts. Cell trajectories were analyzed using methods similar to those described previously (49). To exclude non-motile bacteria, tracks were discarded if the 90th percentile of speed was less than 15 µm/s. The three swimming modes were distinguished based on speed and angular velocity criteria (50, 51).

From individual cell trajectories, we calculated swimming velocities v at each frame and the velocity angular changes ∆θ between adjacent frames. The local maximum of the speed decrease is defined as Δ*v* = max [*v*(*t*_1_) − *v*(*t*_min_), *v*(*t*_2_) − *v*(*t*_min_)], where *t*_min_ is the time at which a local minimum of swimming speed is located, and *v*(*t*_1_) and *v*(*t*_2_) are the closest local maxima located around *t*_min_. A pause or reversal event was identified when Δv was larger than both 1.5 times $v(t_{min})$ and a fixed speed threshold of 9 μm/s. The choice of this speed threshold did not significantly affect distributions of run lengths and pause durations (Fig. S5). Event duration was determined by the condition $v(t)-v(t_{min})\leq 0.4\Delta v$. An event was classified as a pause if multiple peaks of ∆θ with peak values exceeding $2\sqrt{4D_{rot}\Delta t}$ occurred during the event, where $D_{rot}=0.1rad^{2}/s$ is the rotation diffusion coefficient of the cell body, and Δt=1/15s is the time interval between adjacent frames. Events not meeting this criterion were classified as reversals. To calculate directional change between adjacent runs, the last five frames of the preceding run and the initial five frames of the subsequent run were fitted with linear regression. The angle between the two fitted lines was identified as the directional change.

## SUPPLEMENTAL MATERIAL

Supplemental table and figures aem.00597-26-s0001.docx Table S1 and Figures S1 to S5.

Movie S1 aem.00597-26-s0002.avi Flagellar filament dynamics during pause behavior in porous media. Video was recorded at 50 fps and played at 10 fps.

ASM does not own the copyrights to Supplemental Material that may be linked to, or accessed through, an article. The authors have granted ASM a non-exclusive, world-wide license to publish the Supplemental Material files. Please contact the corresponding author directly for reuse.
