# Supplementary material for: c-di-GMP-mediated pause behavior enables Pseudomonas aeruginosa navigation in porous environments
Source: Appl Environ Microbiol. 2026 Apr 27;92(5):e00597-26. doi: 10.1128/aem.00597-26 (PMC13188907; doi:10.1128/aem.00597-26)
Supplement: Supplemental table and figures — Table S1 and Figures S1 to S5. [file aem.00597-26-s0001.docx]

**Supporting Information**

**Table S1.** Strains and plasmids used in this study

| Name | Genotype/Description | Source |
| --- | --- | --- |
| *P. aeruginosa* strains |  |  |
| PAO1 | Wildtype | Lab stock |
| *fliC*^T394C^ | PAO1 containing *fliC* with Thr at 394 mutated to Cys | Ref. 14 |
| *flgZ*^R126A R130A^ | PAO1 containing *flgZ* with Arg at 126 and 130 mutated to Ala | This study |
| *E. coli* strains |  |  |
| DH5α | *F- endA1 glnV44 thi-1 recA1 relA1 gyrA96 deoR nupG Φ80dlacZΔM15 Δ(lacZYA-argF) U169 hsdR17(r_K_^-^ m_K_^+^), λ-* | Invitrogen |
| Plasmids |  |  |
| pCdrA::*gfp* | *gfp* based *cdrA* promoter reporter plasmid; Amp^r^ Gm^r^ | Lab stock |
| pEX18Gm | Gene replacement vector derived from pUC18; Gm^r^ | Lab stock |
| *flgZ*-pEX18Gm | *flgZ*^R126A R130A^ cloned into pEX18Gm; Gm^r^ | This study |
| *flgZ-*pJN105 | *flgZ* overexpression vector in pJN105; pBAD promoter; Gm^r^ Amp^r^ | This study |

**Movie S1.** Flagellar filament dynamics during pause behavior in porous media. Video was recorded at 50 fps and played at 10 fps.


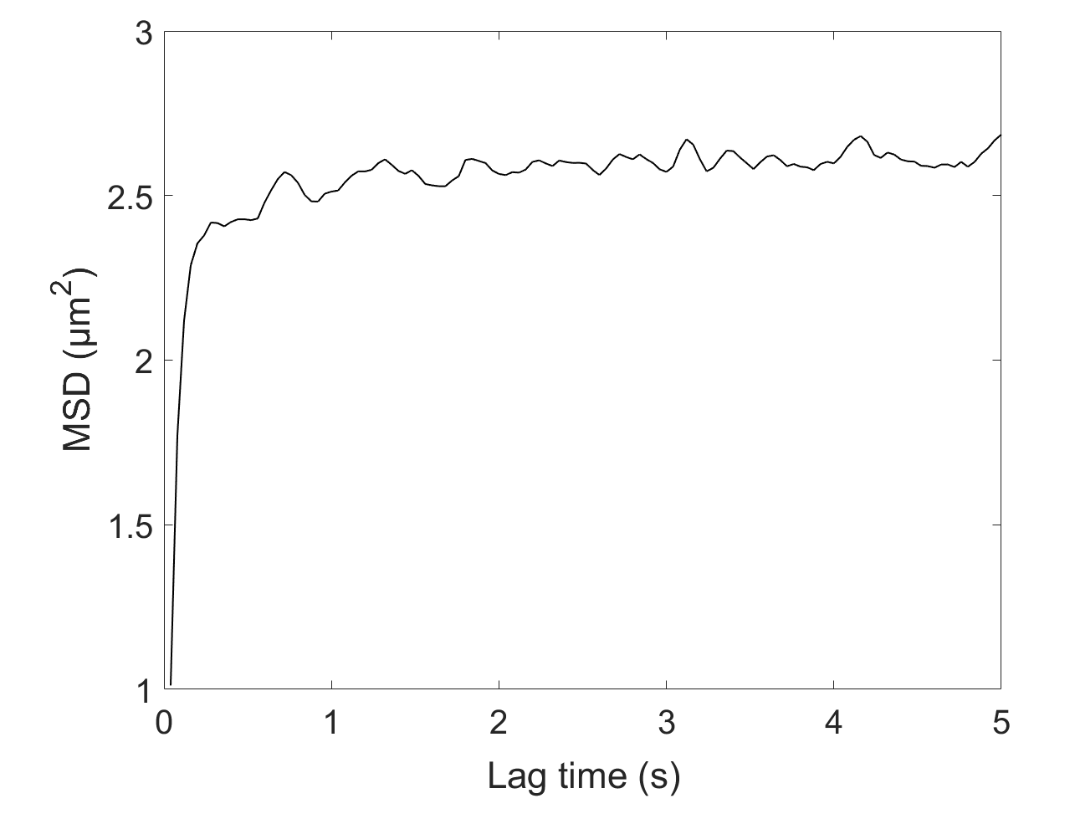


**Fig S1.** Mean squared displacement (MSD) as a function of lag time for an individual 350 nm bead in 0.1% soft agar.


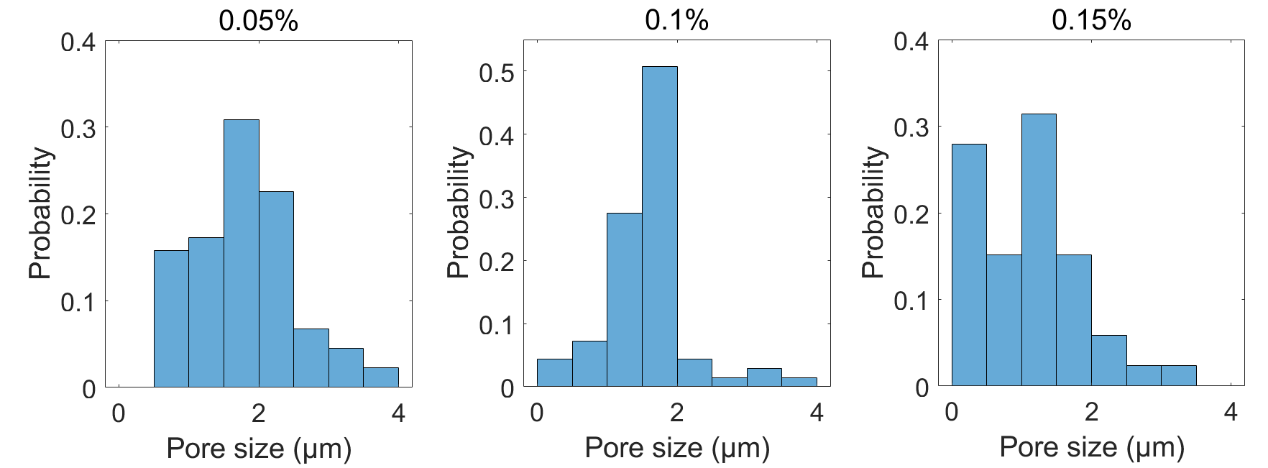


**Fig S2.** Pore size distribution in agar gels of varying concentrations measured using 350 nm tracer beads.


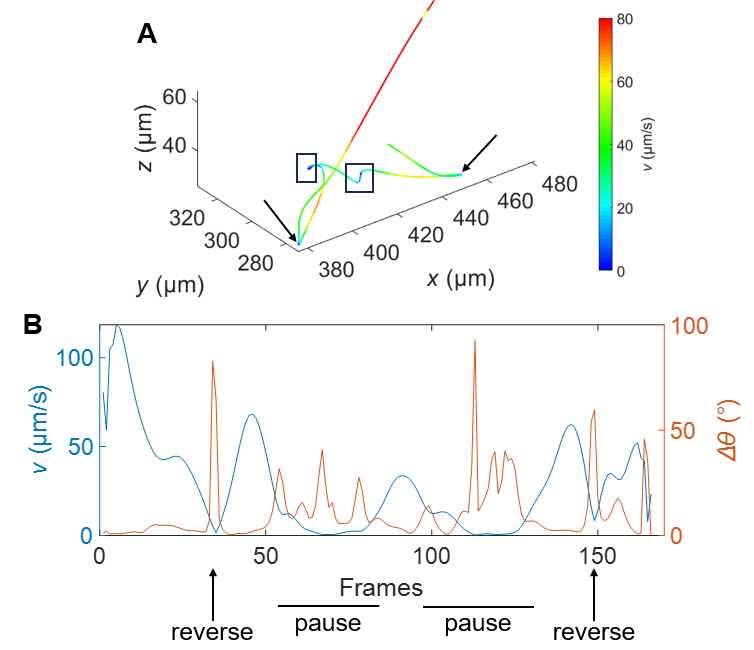


Fig S3. *P. aeruginosa* exhibits the run-reverse-pause swimming pattern in agarose gel. **A**. Representative 3D trajectory of a single bacterium in 0.2% agarose gel. The colorbar indicates instantaneous speed of the bacterium. Arrows indicate reverse events and boxed regions indicate pause periods. **B**. Quantitative analysis of the trajectory in (A) showing instantaneous speed and angular change between consecutive frames as functions of time. Arrows indicate reverse events; horizontal lines denote the pause periods.


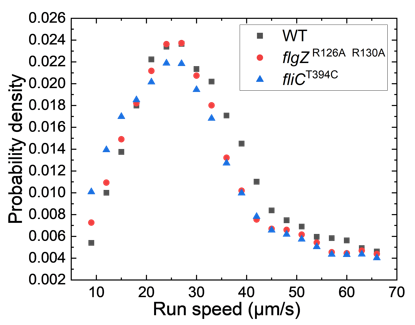


**Fig S4.** Mutations used in this study do not significantly affect bacterial motility in liquid medium.


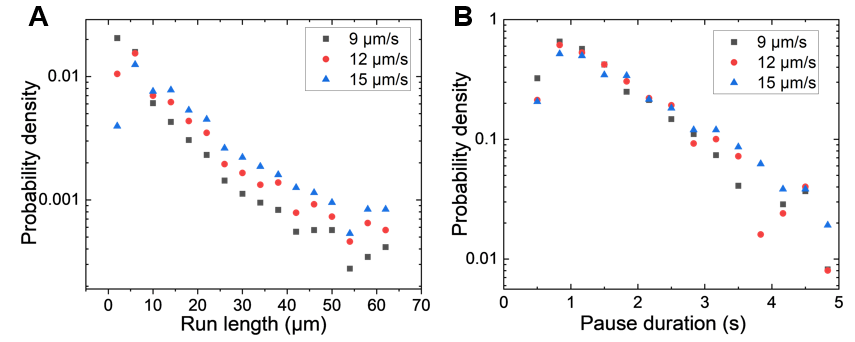


**Fig S5.** The choice of speed threshold does not significantly affect the distributions of run lengths (A) and pause durations (B). Legend indicates the fixed speed thresholds used. The agar concentration is 0.1%.
